# Supplementary material for: Direct observation of strong surface reconstruction in partially reduced nickelate films
Source: Nat Commun. 2024 Jan 9;15:378. doi: 10.1038/s41467-023-44616-x (PMC10774438; doi:10.1038/s41467-023-44616-x)
Supplement: Supplementary file 1 — Supplementary Information [file 41467_2023_44616_MOESM1_ESM.docx]

**Supplemental information of**

**Direct observation of strong surface reconstruction in partially reduced nickelate films**

Chao Yang^1,^ *, Rebecca Pons^1^, Wilfried Sigle^1^, Hongguang Wang^1^, Eva Benckiser^1^, Gennady Logvenov^1^, Bernhard Keimer^1^, Peter A. van Aken^1^

^1^ Max Planck Institute for Solid State Research, Stuttgart, 70569, Germany

* Corresponding author: [c.yang@fkf.mpg.de](mailto:c.yang@fkf.mpg.de)


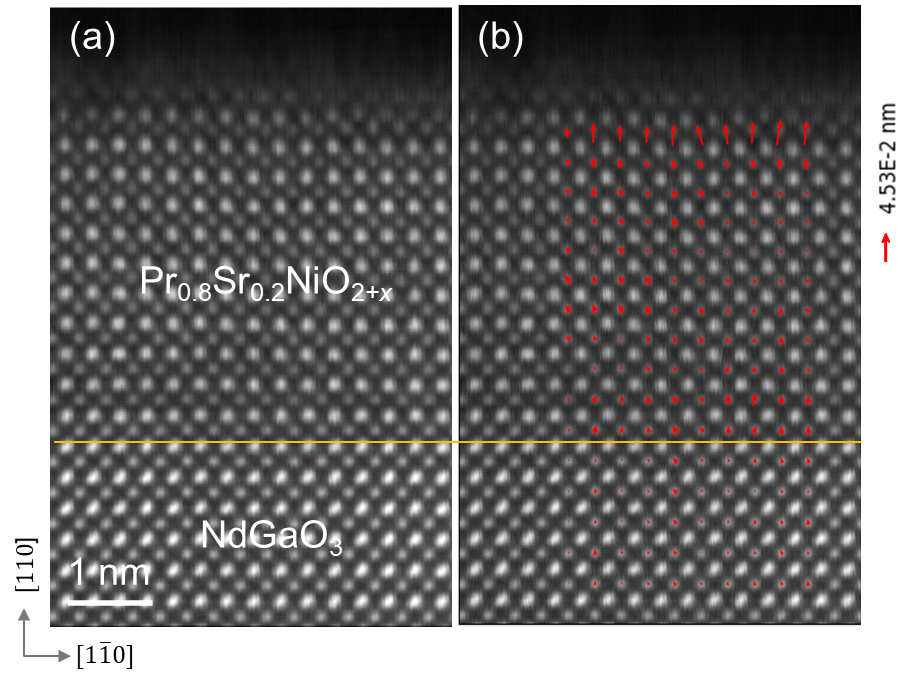


Figure S1: (a) HAADF image and (b) corresponding Ni displacement vector map of the Pr_0.8_Sr_0.2_NiO_2+_*_x_* sample with a topochemical reduction time of 18 h. The red arrows indicate the direction and magnitude of B (B: Ni, and Ga) displacements. The largest displacement of Ni is 0.453 Å.


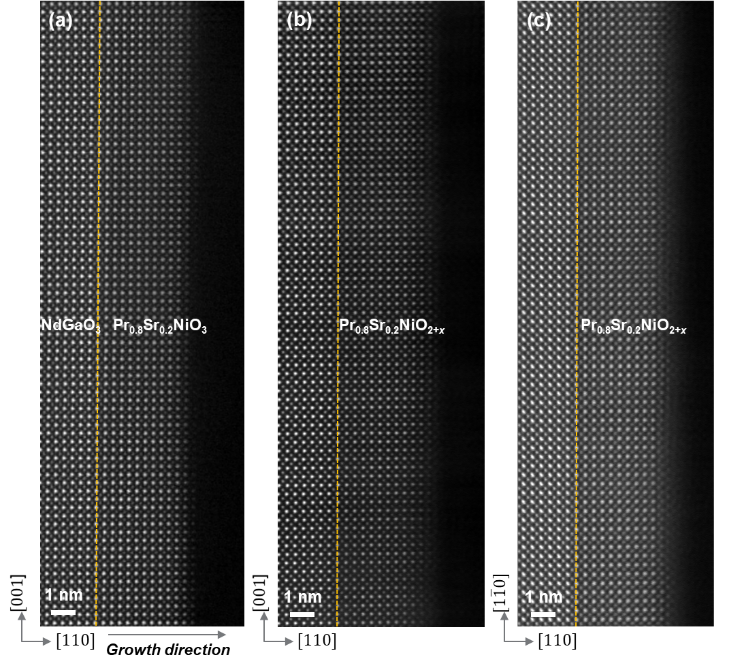


Figure S2: HAADF images of (a) a fully oxide Pr_0.8_Sr_0.2_NiO_3_ film, Pr_0.8_Sr_0.2_NiO_2+_*_x_* films reduced for (b) 6 h and (c) 18 h.


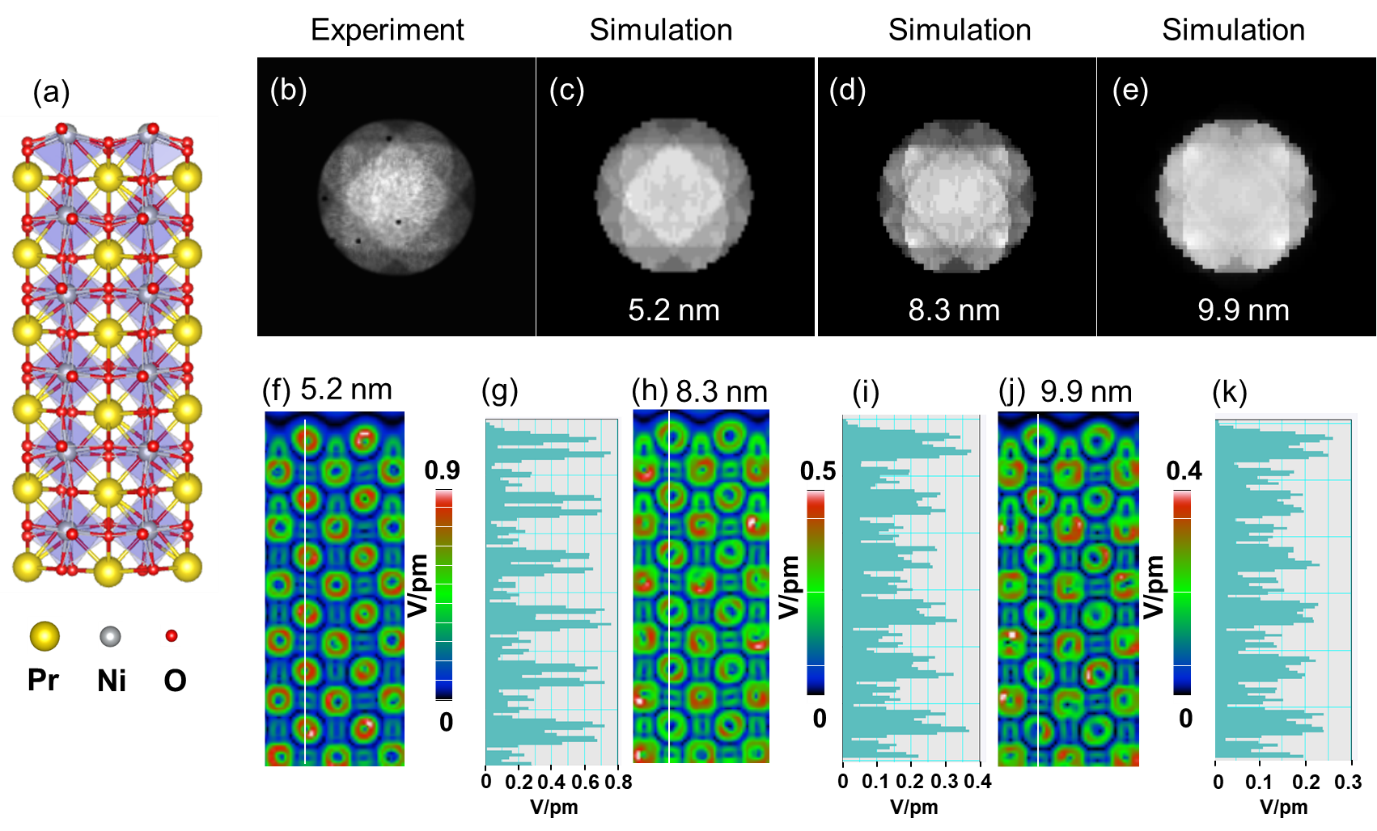


Figure S3: PACBED patterns and electric field maps with different sample thicknesses extracted from the simulated 4D-STEM. (a) A PrNiO_3_ supercell created from the ABF image of a reduced sample with polar distortions at the upper surface. (b) The PACBED pattern extracted from the experimental 4D-STEM. Simulated PACBED patterns for sample thicknesses of (c) 5.2 nm, (d) 8.3 nm, and (e) 9.9 nm. Simulated electric field maps for sample thicknesses of (f) 5.2 nm, (h) 8.3 nm, and (j) 9.9 nm, and their corresponding line profiles of the electric field maps marked with white lines are in (g), (i), and (k), respectively.


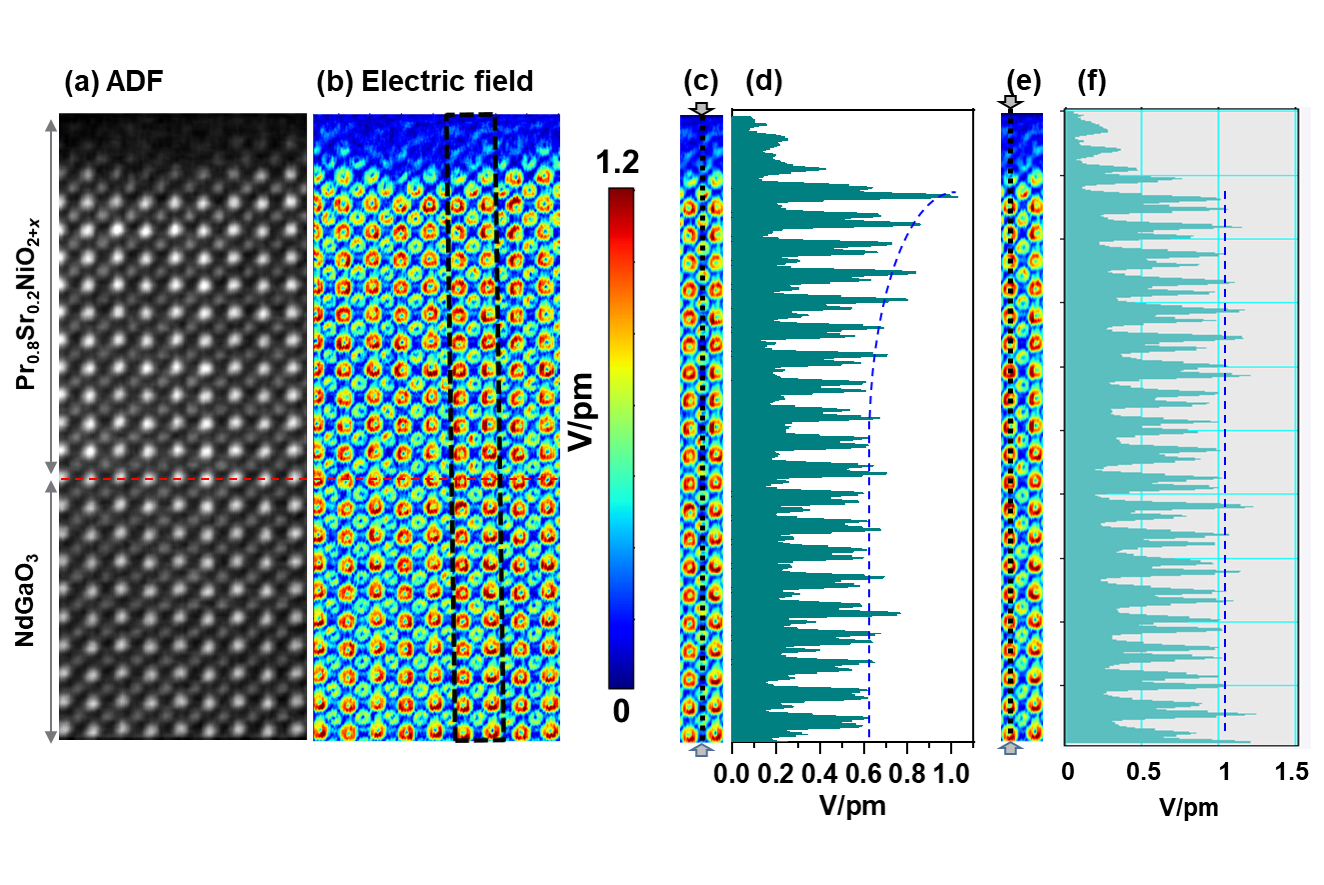


Figure S4: Extracted information from a 4D-data set of the reduced Pr_0.8_Sr_0.2_NiO_2+_*_x_* film. Reconstructed atomic-column-resolved (a) ADF, and (b) electric field images. (c) The electric-field map extracted from the region marked with a black dashed box in (b). (d) The line profile extracted from the region marked with a black dashed line in (c) indicates the changes in the magnitude of the electric field around B (B: Ni, and Ga) atoms. (f) The line profile extracted from the region marked with a black dashed line in (e) indicates the changes in the magnitude of the electric field around A (A: Pr, and Nd) atoms.


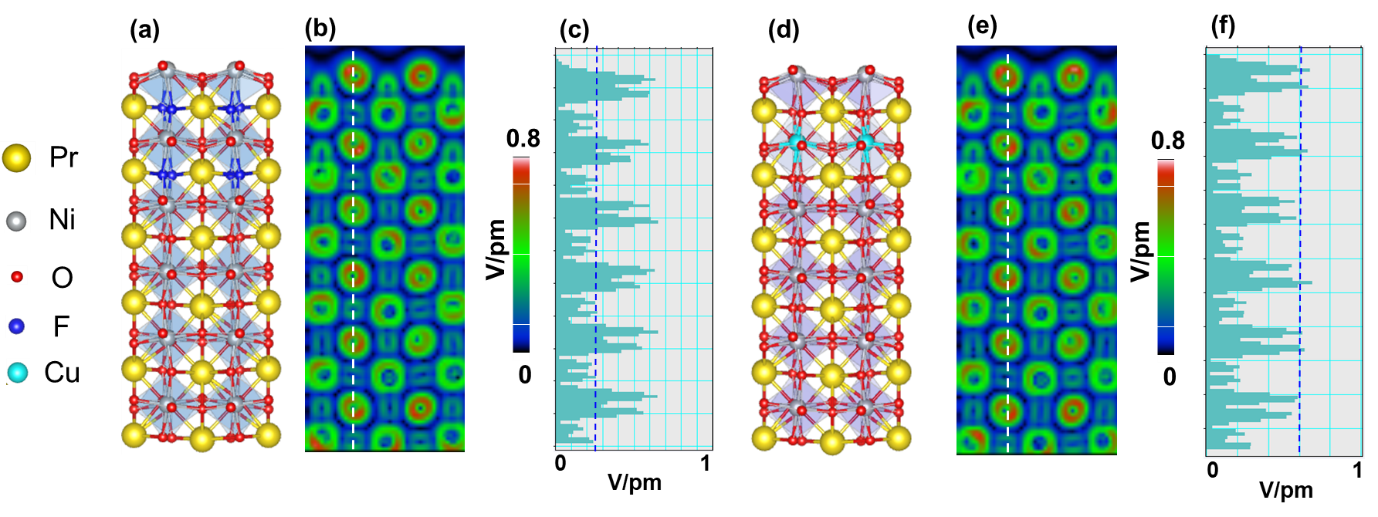


Figure S5: Simulated electric field maps of the samples with different atomic potentials by substituting O and Ni with F and Cu atoms, respectively. (a) A PrNiO_x_F supercell and two apical oxygen layers were replaced by F atoms. (b) The simulated electric field map of the PrNiO_x_F supercell, and (c) the corresponding line profile of the electric field maps marked with white lines in (b). The blue line indicates the field strength of F and O columns. (d) A Pr(Ni, Cu)O_3_ supercell and one Ni layer were replaced by Cu atoms. (e) The simulated electric field map of the Pr(Ni, Cu)O_3_ supercell, and (f) the corresponding line profile of the electric field maps marked with white lines in (e). The blue line indicates the field strength of Ni and Cu columns.


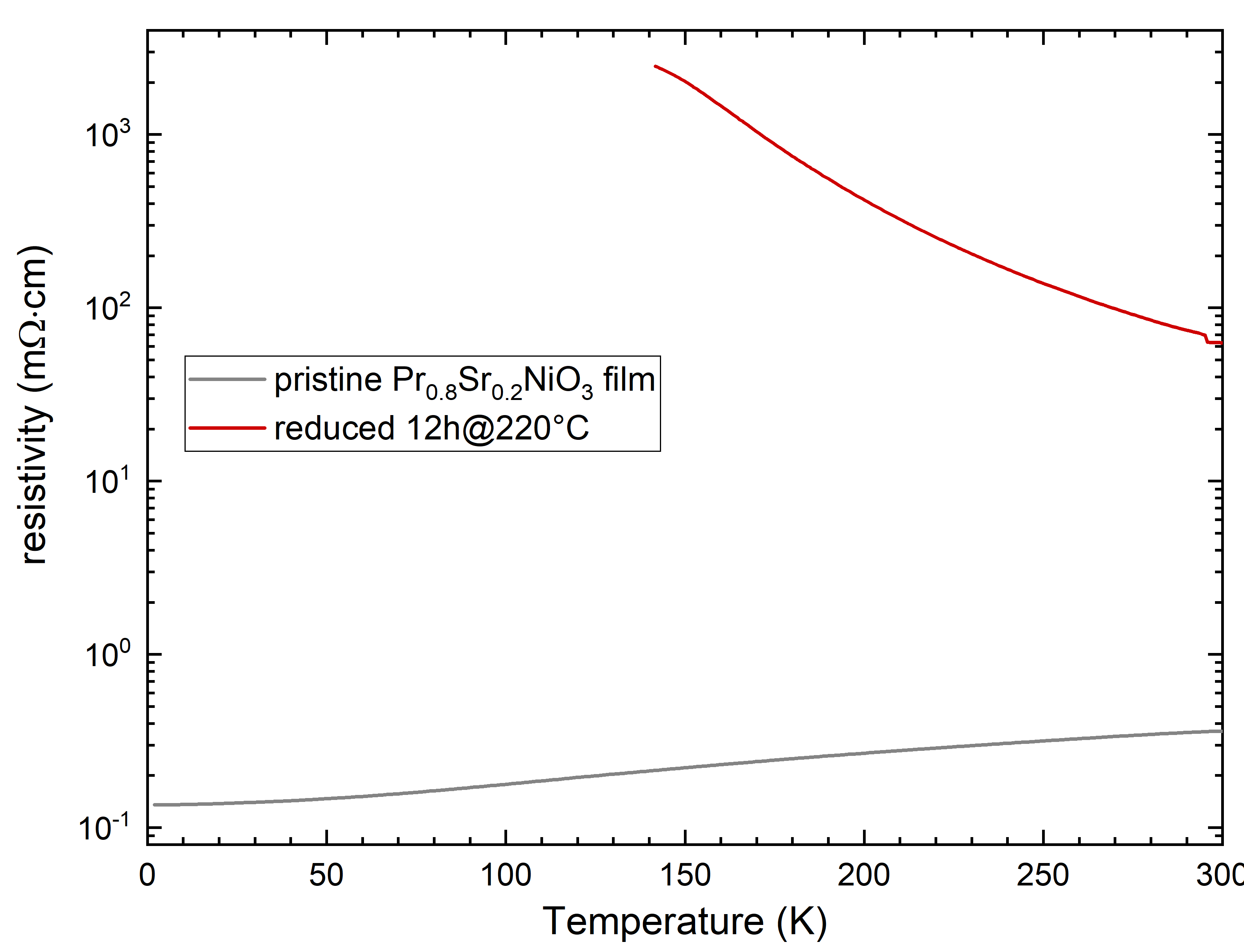


Figure S6: Temperature-dependent resistivity data for the as-grown, pristine perovskite thin Pr_0.8_Sr_0.2_NiO_3_ (black line) and a reduced piece of this sample (red line), measured with a van-der-Pauw 4-point probe geometry in a Quantum Design PPMS.


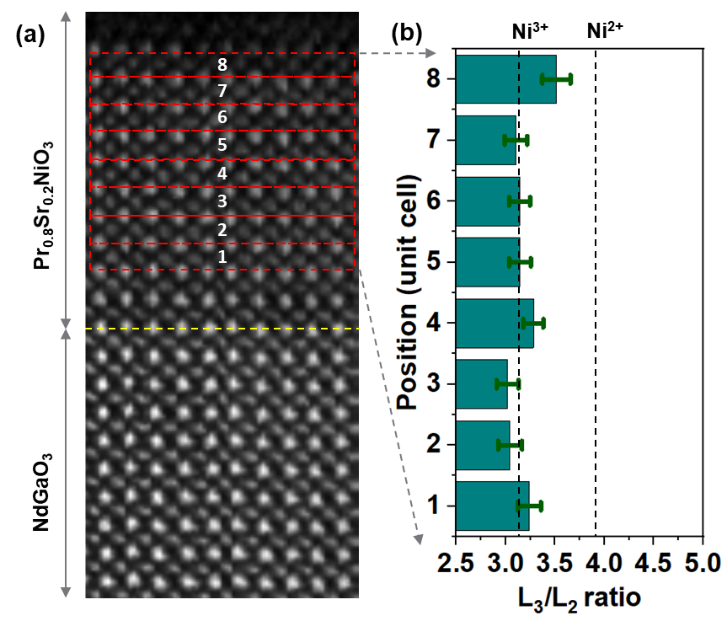


Figure S7: EELS measurement of the Ni-*L*_2_,_3_ edges in the pristine Pr_0.8_Sr_0.2_NiO_3_ film. (a) ADF image for EELS data analyses. (b) Ni-*L*_2_,_3_ white-line ratios extracted from the regions marked in (a).


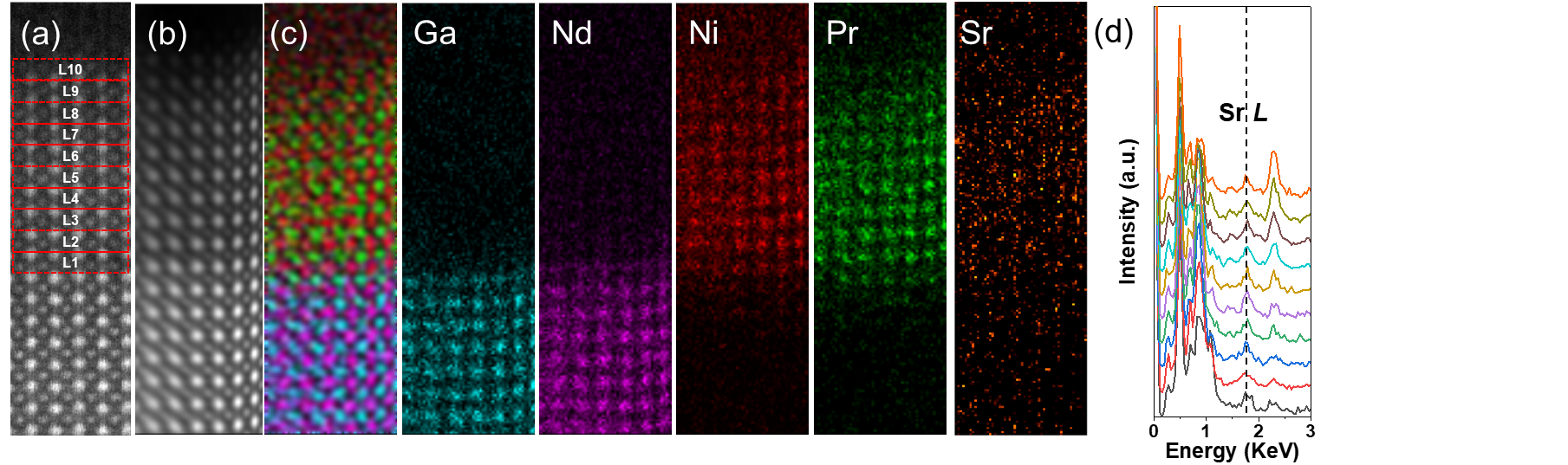


Figure S8: Elemental distribution measured by STEM-EDX. (a, b) HAADF images for the STEM-EDX measurements. (c) Color-coded mapping of Ga (blue), Nd (purple), Ni (red), Pr (green), and Sr (orange). (d) Extracted layer-by-layer STEM-EDX spectra in the Pr_0.8_Sr_0.2_NiO_3_ film.


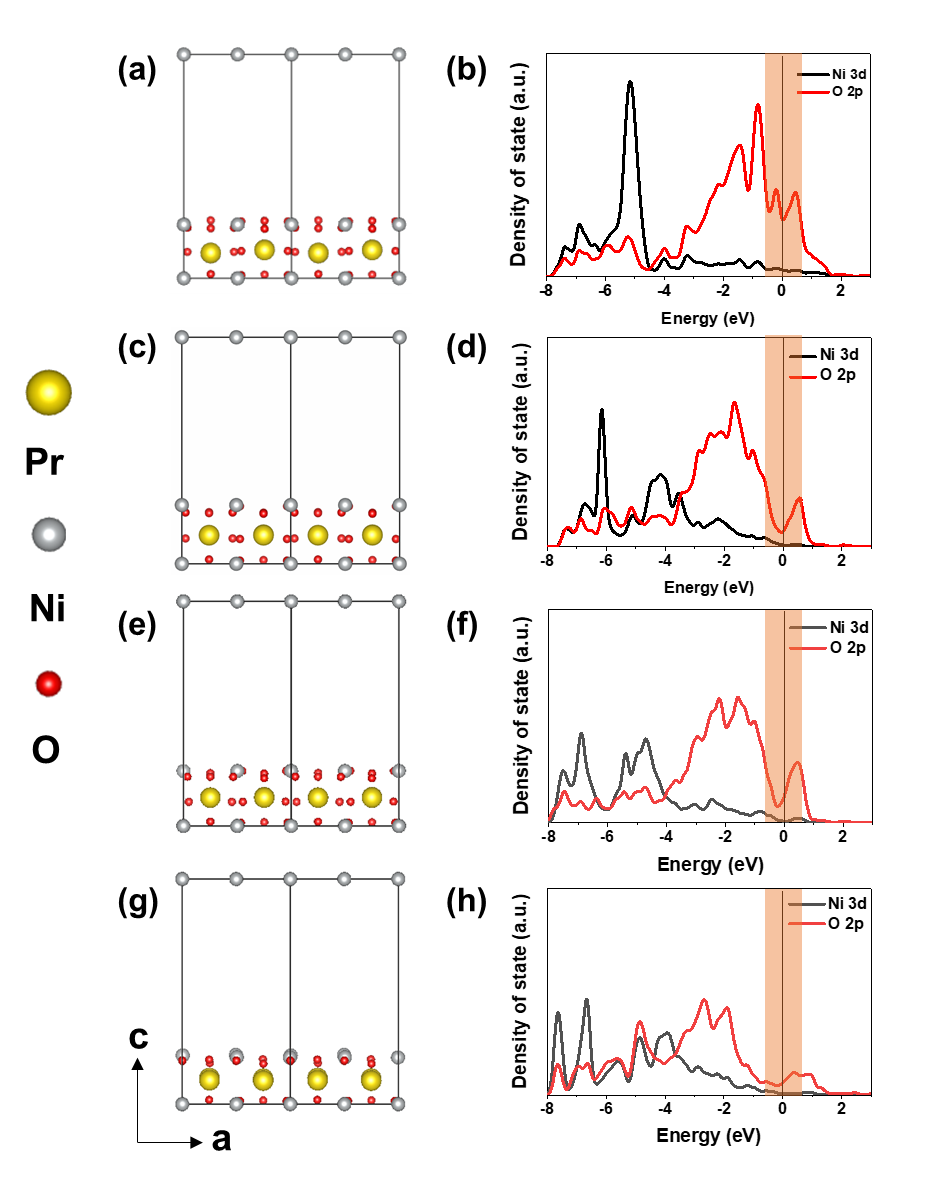


Figure S9 Structural changes and corresponding electronic density of states (DOS) calculated by DFT. (a) Unrelaxed PrNiO_3_ supercell with a [NiO_2_] surface, (c) unrelaxed PrNiO_3_ supercell with a distorted [NiO_2_] surface modified according to the experimental result, (e) relaxed PrNiO_3_ supercell in (c), and (g) relaxed PrNiO_2_ supercell with a [NiO_2_] surface. All structure models have a 10 Å vacuum layer. The corresponding projected DOS plots are shown in (b), (d), (f), and (h), respectively. Orange shadows mark the Fermi level energy range.

Ab initio DFT calculations for the DOS variation at the (110) surface of nickelates were performed using the generalized gradient approximation (GGA) and the Perdew-Burke-Ernzerhof (PBE) functional for exchange correlation, as implemented in the Vienna Ab initio Simulation Package (VASP)^1, 2^. A plane-wave basis set with a cutoff energy of 520 eV is used. The DFT + U method was used with the Hubbard U parameter of 6.2 eV for the Ni 3*d* state^3^. A 10-Å vacuum layer was used for each calculation. The structure optimization was performed with convergence tolerances of 0.01 eV/Å for the maximum ionic force, 5 × 10^−5^ eV/atom for the total energy, and 0.005 Å for the maximum ionic displacement. The VASPKIT code^4^ is used to post-process the VASP calculated data. Figure S9(a) shows the PrNiO_3_ supercell transformed from the PrNiO_3_ orthorhombic unit cell. A NiO_2_ terminated surface was created and a 10 Å vacuum layer was added. Figure S9(b) shows the projected density of states of the O 2*p* and Ni 3*d* orbitals where they hybridize near the Fermi level. Figure S9(c) shows the PrNiO_3_ supercell with a clear surface polar distortion modified according to the experimental result, where there is a Ni-O-Ni buckling structure and the apical oxygen moves away from the Ni atoms due to the electrostatic field at the surface. The Ni-O displacement is set to 0.5 Å, similar to the result in Figure S9(e). The corresponding electronic states decrease near the Fermi level marked by the orange shadow in Figure S9(d), compared to the state of the undistorted structure in Figure S9(b). After structure relaxation, there is a slight change in the surface structure in Figure S9(e), and the corresponding DOS plot in Figure S9(f) is similar. In addition, we introduce the apical oxygen vacancy at the surface layer, forming an infinite layer structure. After structure optimization, we find that there is an obvious decrease in the out-of-plane Ni-Ni distance and a Ni-O-Ni buckling structure in Figure S9(g), which is consistent with the reported result in a similar NdNiO_2_ system^5^. From the projected DOS plot in Figure S9(h), we can see the further decreased orbital overlap between the Ni 3*d* and O 2*p* near the Fermi level, marked by the orange shadow. This reduces the charge transfer capability between Ni and oxygen.

**References**

1. Kresse G, Furthmüller J. Efficient iterative schemes for ab initio total-energy calculations using a plane-wave basis set. *Phy. Rev. B* **54**, 11169-11186 (1996).

2. Kresse G, Furthmüller J. Efficiency of ab-initio total energy calculations for metals and semiconductors using a plane-wave basis set. *Comput. Mater. Sci.* **6**, 15-50 (1996).

3. Jain A*, et al.* Commentary: The Materials Project: A materials genome approach to accelerating materials innovation. *APL Mater.* **1**, 011002 (2013).

4. Wang V, Xu N, Liu J-C, Tang G, Geng W-T. VASPKIT: A user-friendly interface facilitating high-throughput computing and analysis using VASP code. *Comput. Phys. Commun.* **267**, 108033 (2021).

5. He R*, et al.* Polarity-induced electronic and atomic reconstruction at NdNiO_2_/SrTiO_3_ interfaces. *Phy. Rev. B* **102**, 035118 (2020).
